# A qualitative assessment of cleaning and hand hygiene practices at shelters serving people experiencing homelessness during the COVID-19 pandemic, Atlanta, GA – May-June, 2020

**DOI:** 10.1186/s12889-023-16504-y

**Published:** 2024-01-22

**Authors:** Bethlehem N. Besrat, Emily Mosites, Martha P. Montgomery, Amanda G. Garcia-Williams, Emily Trautner, Kristie E. N. Clarke, Brittany Marshall, Cathryn Vassell, Candace Rutt, Shantrice L. Jones

**Affiliations:** 1 Centers for Disease Control and Prevention COVID-19 Response Team, Atlanta, GA USA; 2 Emory University, Atlanta, GA USA; 3 Partners for Home, Atlanta, GA USA

**Keywords:** COVID-19, Hand Hygiene, Homeless shelters, Prevention, Cleaning and Disinfection, Preparadness

## Abstract

**Background:**

Cleaning practices and hand hygiene are important behaviors to prevent and control the spread of infectious disease, especially in congregate settings. This project explored hygiene- and cleaning-related experiences in shelters serving people experiencing homelessness (PEH) during May–June 2020 of the COVID-19 pandemic.

**Methods:**

We conducted qualitative, in-depth interviews by phone with 22 staff from six shelters in Atlanta, Georgia. The interview guide included questions about cleaning routines, cleaning barriers and facilitators, cleaning promotion, hand hygiene promotion, and hand hygiene barriers and facilitators. We analyzed interview transcripts using thematic analysis.

**Results:**

Multiple individuals, such as shelter individuals (clients), volunteers, and staff, played a role in shelter cleaning. Staff reported engaging in frequent hand hygiene and cleaning practices. Barriers to cleaning included staffing shortages and access to cleaning supplies. Staff reported barriers (e.g., differing perceptions of cleanliness) for clients who were often involved in cleaning activities. Barriers to hand hygiene included limited time to wash hands, forgetting, and inconvenient handwashing facilities. Specific guidance about when and how to clean, and what supplies to use, were requested.

**Conclusion:**

During the early months of the COVID-19 pandemic, shelters serving PEH in the Atlanta-metro area needed resources and support to ensure sufficient staffing and supplies for cleaning activities. As part of future pandemic planning and outbreak prevention efforts, shelters serving PEH could benefit from specific guidance and training materials on cleaning and hand hygiene practices.

## Background

On any given night, more than 326,000 people in the United States experience sheltered homelessness [1]. Over 11,000 U.S. community housing and homeless shelters provide services such as transitional housing, emergency housing, and basic necessities [2]. In 2021, 157,397 people were employed in the Community Housing and Homeless Shelters sector in the United States [2], with recent annual growth of almost 3% in this sector [2]. Like other congregate settings, shelters serving people experiencing homelessness (PEH) are at a high risk for infectious disease outbreaks. Shelters have experienced outbreaks of infectious diseases like tuberculosis, severe acute respiratory syndrome (SARS), hepatitis A, and SARS-CoV-2, the virus that causes coronavirus disease 2019 (COVID-19) [3–6]. In a meta-analysis of studies conducted in several countries between January–May 2020, researchers found that 15% of staff working in homeless shelters were infected during SARS-CoV-2 outbreaks in the shelters they worked in [6].

Water, sanitation, and hygiene (WASH) is often promoted as part of the response to infectious disease outbreaks. This includes cleaning practices and hand hygiene, which comprises washing hands with soap and water or using an alcohol-based hand sanitizer. These are important behaviors in preventing and controlling the spread of many infectious diseases [7, 8]. Previous literature has found that lack of guidance, insufficient training, and difficulty obtaining infection control supplies were barriers to implementing infection control measures in homeless shelters and other community settings [4, 5, 9]. Homeless shelter staff and health care providers have also described staffing constraints as barriers to infection prevention and control [10, 11]. However, little is known about WASH practices in shelters serving PEH in general and during infectious disease outbreaks.

**Fig. 1:**
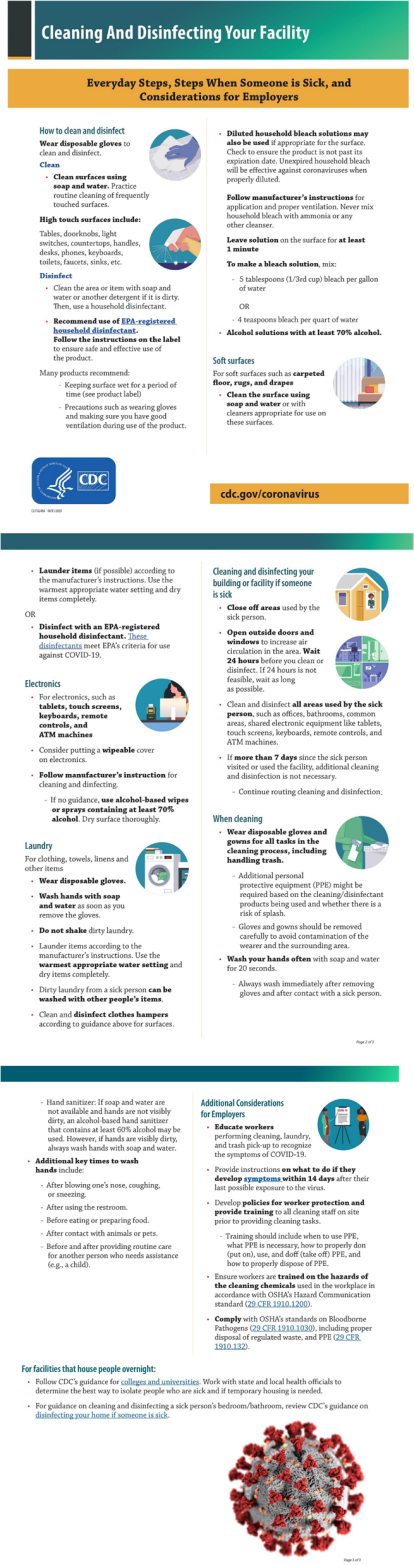
04/01/2020 CDC cleanding and disifnection guidance for facilities

In Spring 2020, CDC developed cleaning and disinfection guidance for general facilities to promote COVID-19 prevention (see Fig. 1) [12]. At the time, much was unknown about the key ways SARS-CoV-2 spread. Non-pharmaceutical interventions such as cleaning and disinfection as well as hand hygiene were promoted during this time. However, little was known about shelters experiences with these recommendations and how they were being implemented. The purpose of this project was to: 1) describe hand hygiene- and cleaning-related experiences and barriers with staff in homeless shelters during the COVID-19 pandemic, and 2) assess preferred communication strategies for shelter staff. The findings from this project can inform areas for staff and facility-level interventions. Additionally, the results of this project can inform future outbreak preparedness and pandemic planning for shelters and facilities serving PEH.

## Methods

We conducted in-depth, qualitative telephone interviews with staff and administrators from six shelters in Atlanta, Georgia. Atlanta was selected as the project site due to the project team’s existing relationships with homeless service providers. We used purposive convenience sampling to identify participating shelters. At each shelter, one administrator and three to four staff were interviewed. Eligibility criteria for staff or administrators to participate were as follows: (1) ability to speak and read English, (2) 18 years of age or older, and (3) serving in a staff or administrator capacity for at least six months.

One member of the project team led the interviews using a semi-structured interview guide. Field notes were taken during the interview by a member of the project team serving as a non-participatory notetaker. The interview guide included questions about cleaning routines; barriers and facilitators to cleaning and disinfection; cleaning promotion; shelter-led hand hygiene promotion; and hand hygiene barriers and facilitators of staff. At the end of the interview, participants were shown the current CDC cleaning and disinfection guidance (Fig. 1) and were asked about preferred communication regarding hand hygiene and disinfection for shelter residents and staff. On average, interviews took 35 min, and each participant received a $25 gift card as compensation for their time. All interviews were audio recorded and the audio was transcribed verbatim for analyses.

We used inductive and deductive coding to develop a codebook for analysis. A team-based coding approach was used to code transcripts. Two researchers reviewed and coded each transcript together. One researcher shared the screen with the transcript visible to the second researcher and took turns reading the transcript aloud. All codes were discussed until 100% agreement was achieved. This activity was determined to be non-research and was conducted consistent with applicable federal law and CDC policy. ^1^ MAXQDA 20.0 (Verbi Software) was used for data management [13].

## Results

A total of 22 interviews were conducted from May through June 2020 with homeless shelter staff and administrators from six shelters. Shelters ranged in size from 20 to 450 beds and served various populations, such as men, couples, veterans, women and children, and families. Shelter types included low-barrier shelters (facilities without extensive requirements for use), emergency shelters (temporary shelter), night shelters (temporary night-by-night lodging), transitional housing (long term temporary lodging with intensive services), day shelters (where individuals can spend time during the day), or a mixture. Respondents worked in various roles such as a resident manager, vocational training coach, supervisor, director, administrator, or case manager.

### Cleaning and disinfection

Respondents reported use of existing cleaning and disinfection protocols, such as those for tuberculosis (TB) prevention, that were established prior to COVID-19. Respondents described using routine cleaning schedules that included cleaning floors, wiping down high-touch surfaces, cleaning electronics, regular bathroom cleaning, doing laundry, and routine “deep-cleaning” (Table 1).

**Table 1:** Themes related to cleaning and disinfection practices, barriers, facilitators, and health promotion among in-depth interview respondents

**Environmental Cleaning**		
**Theme**	**Examples**	**Quotes**
Cleaning and Disinfection activities	• Cleaning protocols already in place • Wiping down high-touch surfaces • Cleaning floors and walls • Sweeping and mopping • “Deep-cleaning” by removing furniture from rooms to clean all spaces	• “And so we just utilize our daily TB protocols, which involves, you know, and our habitability areas. We, we wipe down all surfaces in our habitability areas, in our dorms. Uh, in the showers we do regular cleaning, pressure washing of the showers. We mop all the floors and wipe down all the walls daily with, uh, with a bleach combination that we, that we purchase. Um, we wipe down all high touch areas, uh, clean all ventilation, uh, and this is what we do daily as part of our TB protocol that we just, we just rolled over into, uh, COVID, uh, 19 protocol.”
Person Responsible	• Clients • Volunteers • Maintenance staff • Non-maintenance staff	• “There's always staff. It, it, it's a combination of staff and clients, together.” • “Yes, our clients clean the areas that- the- the community areas, the bathrooms, and then of course their rooms, twice a day. Well, not their rooms twice day, but we also have a cleaning company that comes in once a week.”
Cleaning Routine	• Cleaning follows standard schedules or routine • Before a new client comes in and after a client leaves • Every two hours • Three times per day (morning, afternoon, evening) • Weekly “deep clean”	• “We clean um, and disinfect three times a day, at 8:30 in the morning, at one in the afternoon, and at six at night. We clean all the highest trafficking areas, which is the main entry way, the atrium, where the ladies hang out at times and the cafeteria where they eat their lunch, uh, aisle chairs, tables, uh, anything that is being touched, or we feel that is being touched by a client or either staff. We make sure that, um, we get in and clean it and disinfect it three times a day, including the restrooms and also our play area outside for the children.”
Cleaning Products Used	• Disinfecting wipes • Unspecified sanitizing spray • Hospital grade chemicals/cleaner • Specified sanitizing spray (Fabuloso, Lysol, Gammim-14)^a^ • Alcohol • Bleach • Glass cleaner • Lemon or pine cleaner	• “Uh, we use um, bleach. We use um, Lysol. There's some products that we received from one of our partner agencies, I cannot tell you the name of the products, um, but um, mainly disinfectants are products to keep everything clean and sanitized.” • “We use a disinfectant that the hospitals use called um, Neutral Fresh and bleach, and we have some disinfectants spray that we use also.”
Changes to Cleaning Routine	• Increase in cleaning frequency • Focus on high-touch areas that haven’t been historically prioritized in cleaning (e.g., doorknobs, light switches)	• “I would say disinfecting the, the common areas like the, the light switches, and sanitizers (laughs), and the doorknobs. We weren't doing that before. We were cleaning, you know, you know, with the, had the regular chores but we weren't doing that until the Coronavirus.”
**Cleaning and Disinfection Promotion**		
**Theme**	**Examples**	**Quotes**
Education	• Training on how, where, when to clean, use of personal protective equipment, and daily protocols • Talks on how and why there is a need to keep the facility clean • Coach on how to clean • Community classes about hygiene	• “Yes. Well, all of that takes place. And we have o- our, um, staff meeting, um, it was stressed very much then, uh, you know, about how we have to make sure that we, uh, keep everything in order and- and everything disinfected and clean and- and it was, it was, it was just, um, we, and we also had, um, Fulton County department of health come in, they talked to us.”
Format	• Posters • Flyers • In-person trainings • Discussions during staff meetings • Orientation sessions for clients • Community meetings • Virtual trainings or webinars • One-on-one discussions or trainings • Emails	• “We ha- have sent out emails, pamphlets, um, encouraged them to sit on Webinars about it.” • “Well, during staff meetings we address the cleaning and we've always done that and also when we have new clients, we have orientations, so we orient them on the- the expectations and the procedures and the policies.”
**Cleaning and Disinfection Barriers**		
**Theme**	**Examples**	**Quotes**
Individual Barriers	• Severe mental and physical health problems make cleaning hard • Keeping clients focused and understanding of importance of cleaning • Apathy, lack of engagement or motivation • Different ideas of clean • Different cleaning styles • Complaints about smell of cleaning products	• “And so it, sometimes, it's the challenge just to tell gentlemen, "Oh, yeah you know, I'm, I know you're tired of doing the same thing, and yeah, it may be repetitive or whatever, but we have to do it, and it's important that we do it." And just keeping it, keeping them focused is, is, is to me, one of the challenges to uh, to making this a such, you know, a successful operation… “ • “… we can motivate them. I mean, I think a lot of guys don't want to have skin in the game. They just don't want to participate, but that, that's part and parcel to their entire, to their entire life. And so, um, we have to, you know, interact with them and create relationship that, you know, that overcomes some of that apathy. Of course, there's some guys that are just apathetic, you know, and not, not want to participate at all.”
Access	• Cleaning supplies and chemicals • Preferred supplies not available • Gloves • Gowns for cleaning	• “Availability of products has been, it… I'm sure you hear this from everybody you talk to right now. It's hard to get stuff.” • “No, I think the only other thing would be having more access to, like, Lysol type wipes or disinfectant spray. So like, there's not enough for, so like if staff, so like something we talked about the other day was okay, wait, this is great, the clients are deep cleaning three times a day. Are staff cleaning their areas? Nope. Um, and so it's like, okay, if each staff had a bottle of disinfectant spray. Or had, you know, a container of Lysol wipes, they would probably be more apt to actually, um, clean more.”
Capacity	• Large number of clients rotating through • Understaffing or lack of volunteers to keep up with increased cleaning frequency • Individuals who can reliably do good cleaning	• “… most of our cleaning is done… by residents. Then, if we don't have the residents to do it then with us trying to social distance with staff, we don't have the amount of staff that we used to and so, people are being pulled into different roles. And so it might be on me certain day to see clients and then also make sure that the lobby has been disinfected. So, it's just, I guess that would be the biggest challenges that um, because we're still trying to, you know, service our clients and then also we have to adhere to the obligations and the requirements for sanitation.”
**Cleaning and Disinfection Facilitators**		
**Theme**	**Examples**	**Quotes**
Oversight	• Monitoring by managers or staff • Discussion with clients about why cleaning didn’t occur or wasn’t done according to protocols • More reinforcement of cleaning behaviors • Tracking logs	• “Keeping everybody informed and then going back and checking areas, check, check, check, check. To make sure that we are staying in compliance. And then being brutally honest with ourselves, with um, where we're succeeding, where we're having difficulty and where we uh, may have missed the mark or missed something that we now need to address.”
Other	• Put positive spin on cleaning activities • Give people ownership • Cleaning built into daily activities and rhythm • Increasing awareness of why cleaning is important • Clients being willing to help with cleaning • Working together as a team, everyone pitching in	• “… depending on the client and depending on the area, we've definitely, um, moved people around as we've noticed their enthusiasm or lack thereof of cleaning to probably less high traffic areas.”
Access	• Access to supplies needed	• “Um, we also have cleaning products, um, in the kitchen area and in the bathroom area. So if someone went in and took a bath, they know that they have to clean behind themselves as well. So they always have an abundance of products, you know, so they never run out. That's a good thing. I've not seen them run out of toilet tissue, paper towels, bleach solution, um, soap or the dish washing detergent, any of that. They've never ran out.”

^a^The use of trade names is for identification purposes only and does not imply product endorsement by the CDC

In response to the pandemic, COVID-19 specific policies were incorporated into existing protocols. For some respondents, these policies were implemented due to CDC Cleaning and Disinfection guidance (Fig. 1). Respondents who were familiar with the guidance described implementing procedures and posting parts of the guidance around the facility. Of those unfamiliar with the CDC guidance, respondents mentioned being familiar with the concepts included in the guidance and implementing many of the recommendations included in the guidance such as cleaning electronics and high-touch surfaces prior to seeing the guidance. Cleaning and disinfection activities occurred several times a day. For example, one shelter cleaned high-touch surfaces every two hours and another shelter cleaned three times a day (morning, afternoon, evening). There was a particular focus on surfaces frequently touched by multiple people, such as doorknobs and light switches. Participants reported that these surfaces had not been prioritized for frequent cleaning prior to the COVID-19 pandemic.

Respondents described using a range of cleaning and disinfection products including solutions and sprays. The products described were commercial-grade, industrial strength, or hospital-grade disinfectants and solutions. Respondents described making a bleach solution but mentioned that they did not mix any other chemicals or products together to make a cleaning solution.

Multiple individuals played a role in shelter cleaning. This included shelter residents (clients), volunteers, maintenance staff, and non-maintenance staff. In some shelters, respondents noted a decline in volunteers being able to help with cleaning practices due to COVID-19 protocols limiting personnel admitted to the shelter site and a decrease in volunteers during the pandemic. This shifted responsibility for cleaning onto staff or both staff and clients. Client roles in cleaning and disinfection varied from shelter to shelter; some clients were expected to clean as in-kind support during their stay, others received job training via their cleaning activities, and some volunteered to assist when there were insufficient staff to engage in frequent cleaning.

Strategies to promote cleaning and disinfection practices in shelters included cleaning protocols and promotional materials sent out by shelter leadership via email. Respondents noted that at the start of the pandemic, this approach was helpful, however over time they received an overload of information and expressed difficulty keeping up with the information. Other promotion strategies included posters, flyers, meetings, and webinars. Posters and flyers served as a reminder of key cleaning practices, while meetings were used to discuss cleaning approaches and issues with cleaning.

To ensure that staff and clients understood cleaning protocols and practices, respondents reported receiving in-person or virtual training on cleaning practices and protocols. Generally, staff did not say who provided trainings; however, of those that reported receiving trainings, the local health department, a Federally Qualified Health Center, maintenance leadership, or human resources led the trainings. Staff in turn trained the clients who supported cleaning activities. Client trainings were offered frequently due to high client turnover.

The main barriers to performing cleaning and disinfection included individual factors, access to supplies, and sufficient staffing capacity. Individual factors included attributes of clients or staff that made cleaning a challenge: for example, serious mental or physical health problems impacted some clients’ ability to support cleaning efforts, and perceptions of cleanliness standards and optimal cleaning techniques differed among people within a shelter. Additionally, it was difficult to relay cleaning information to clients who were unable to speak English, had low literacy, or were illiterate. Other barriers included staff and clients not following cleaning supply directions, using too little or too much cleaning product, lack of consistency with cleaning protocols, space to clean in occupied areas, or getting clients motivated to clean. An uncommon barrier was lack of access to cleaning supplies as respondents had access to cleaning supplies and utilized established cleaning routines.

Increased oversight of cleaning activities, adequate access to supplies, and established routines made it easier to complete cleaning activities. Oversight involved staff doing walk-throughs of the facility and checking product supply levels and how different areas were being cleaned.

### Hand hygiene

Staff respondents reported washing hands with soap and water, using hand sanitizer, and using gloves (Table 2). As a result of the COVID-19 pandemic, staff reported increasing the frequency of hand hygiene behaviors. Common times for handwashing included before and after handling food or after using the restroom. Respondents typically used hand sanitizer when walking into or out of the shelter, before eating, and periodically while working at their desk or office. Several respondents mentioned preferring to wash their hands compared to using hand sanitizer, but because of time constraints and restroom location, respondents described relying on hand sanitizer more.

**Table 2:** Themes related to hand hygiene practices, barriers, facilitators, and health promotion among in-depth interview respondents

**Hand Hygiene Practices**		
**Theme**	**Examples**	**Quotes**
Hand hygiene behaviors	• Washing hands with soap and water • Hand sanitizer use • Wearing gloves	• “Washing the hands, uh, and wearing gloves and uh you know, when, wh- when, folks see that, a lot of times you see people follow, follow in step with that.”
Key moments hand hygiene occurs	• Before handling food • Walking into or out of the facility • Before and after eating • After using the restroom	• “We, we washing our hands at all times, everybody is. You touch something, you know, you know you gotta wash your hands. We don't let the clients use the same utensils. Like when you're being served, we serve them. So we're really the only ones who use that particular utensil.” • “More. Everybody is more, you know… Anybody. We, we wash our hands, you know, like before we eat, after we use the bathroom. We know everybody's more conscious. You wash your hands constantly. If you cough, you're washing your hands. If you touch your face, you're washing your hands. If you touch the counter, you know to hit the hand sanitizer. You know, everybody's doing that more than we would normally have done it due to COVID-19.”
Changes to hand hygiene	• Changes to HH • Increase of washing hands and using hand sanitizer • Less hand shakings, hugs, or high five	• “Well, I see, uh, I see us washing our hands more frequently as well. And then, uh, then using gloves.” • “… and just no interaction in regards to handling hands, uh, like handshakes and stuff like that. No hugs.”
**Hand Hygiene Promotion**		
**Theme**	**Examples**	**Quotes**
Format	• Posters • Flyers • Videos • In-person trainings • Meetings	• “We have posters up, like, at, at the sinks in the bathroom.” • “Well, I mean, we, we do, you know, you put up the posters and you have the training, and you know, we have the little video snippets that we have.”
Hand sanitizer dispenser locations	• Entrance/exit of the facility • Outside the restrooms • Offices • Dining room entrance • Common areas	• “Yes. Okay. So we have sanitizers at the front door, the backdoor, the dining hall, outside the restroom, inside the rest, uh, restrooms. Um, and then we also have gloves.” • “Yeah, no, we have added, um, more, um, dispensers for hand sanitizer… um, in the building. Um, and, like, we've added them in the, like, in new places that we think would be more helpful. Like, when you come off an elevator, when you go on the elevator, when you right come in the door, whatever door you come in. Um, and more on each floor. So, we add- added access to that.”
**Hand Hygiene Barriers**		
**Theme**	**Examples**	**Quotes**
Multiple barriers	• Barriers include: • Remembering to practice hand hygiene • Limited time • Limited access to hand hygiene products • Location of bathrooms • Limited handwashing facilities • Unable to leave hand sanitizer with clients due to high alcohol percentage	• “Just getting up and just accessibility to the bathrooms. I mean it's not [inaudible] per area. It uh, it could be a long walk for some, more so than others. Uh, we have, this was a, this was designed to be an all male facility, but we've, course we've had uh female staff here over the last four or five years. So those combinations had to be created so. It's not easy for everyone just to walk across the hallway, or take a couple of feet here or there to wash their hands. So I guess, again, it's accessibility and just that reminder that something needs to be done more often than usual.” • “Um, just lack of, so like one of the main places that we hang out in the administrative office, there's no sink anywhere close by.”
**Hand Hygiene Facilitators**		
**Theme**	**Examples**	**Quotes**
Access	• Access to products	• “Well, I mean, the biggest thing you can do for staff is provide the tools to do the job… and so if I want them to use hand sanitizer, I have to make sure they have abundant amount of hand sanitizer.”
Motivation	• Positively motivating each other	• “And then we just really, we t- We just kind of make fun of, uh, you know… Make jokes, like, hey did you wash your hands? Don't talk to me, don't touch me until you wash your hands. Stuff like that.” • “But when you, you look at it as, ‘Hey, let's join together to keep us safe and keep your family safe,’ and spin it from a positive perspective, then I notice that most people were compliant, not because you said you had to do it. More because, ‘Hey, I need to, I need to protect myself and my family. You know, I need to take care of myself, you know’."
Hand sanitizer locations	• Placing hand sanitizer dispensers throughout facility	• “So, we've posted that. Um, and then we just talk about it. We have sanitizer posted e- everywhere so they can always, um, you know, do the sanitizer, uh, pump thing. Um, our, our, uh, CDC director has given us bottles of sanitizer. So, we promote that as well.”

Strategies to promote hand hygiene behavior among staff and clients included sharing or posting posters or flyers of when hands should be washed and placing hand sanitizer dispensers in several locations to serve as physical reminders to frequently clean hands. Other promotional methods included videos, trainings, and reviewing proper hand hygiene methods and importance of hand hygiene during staff meetings. Respondents mentioned that washing your hands for at least 20 s, keeping hands away from the mouth and eyes, and how to use hand sanitizer were emphasized during staff meetings/trainings. A few respondents desired signs encouraging hand hygiene to post at their shelters.

Staff generally did not perceive barriers to implementing hand hygiene behaviors. Among staff who expressed having barriers, primary barriers included difficulty remembering to practice hand hygiene, insufficient time, limited access to hand hygiene products, bathroom location, or inadequate hand hygiene facilities. One respondent also noted it was not possible to leave alcohol-based hand sanitizer out in shelters due to concerns for clients with alcohol use disorders.

Staff’s facilitators to frequent hand hygiene included access to products, positively encouraging hand hygiene behaviors among each other, and established routines. The availability of hand sanitizer dispensers and bathrooms being stocked with soap and paper towels facilitated hand hygiene behaviors. Since the start of the pandemic, respondents described an increased awareness to practice hand hygiene throughout the day, making it a routine behavior.

### Guidance communication preferences

Respondents reported a desire for more guidance on the recommended frequency of cleaning and disinfection; how to clean and disinfect specific objects (e.g., leather furniture, toys, electronics); how to clean donated food, furniture, or clothes; and a more concise list of recommended cleaning and disinfection supplies (Table 3). Additionally, respondents wanted guidance on using gloves safely, the disposal process of personal protective equipment, and how often to clean soft surfaces.

**Table 3:** Guidance Communication Preferences

**Guidance Information Needs**		
**Theme**	**Examples**	**Quotes**
How to clean specific items	• Leather furniture • Toys • Electronics • Donated food • Donated furniture • Donated clothes • Soft surfaces	• “…definitely [more instructions] for [cleaning] leather furniture because I don't see guidance concerning that”
When to clean items	• Including information on how frequent items should be cleaned	• “I think that the, the electronics surface 'cause you're using them all the time, it's like what is the frequency about what you should do this?” • “Um, so the only question I have, like, which I'm confused about, is when it's talking about like for soft surfaces to clean. Like, is this just like, so I understand how often or when to clean high touch surfaces. As far as the soft surfaces, like, is this just a if you spill something on it? Or is this something that should be done on a regular basis? How frequently?”
Cleaning products	• Including information on what cleaning and disinfecting products to use • Ideas on how to store products and make it more accessible in large facilities	• “…maybe giving an example of EPA-registered household disinfectants because I don't think … everybody know what EPA, um, registered household disinfectant is.” • “I guess maybe just storage, or how to make it more accessible to use. Um, or maybe ideas about how to do that. Like I said, we keep… all these items in a secure place. But I think that'd be key if there was some creative ways that you could create cleaning stations where it's easy or more accessible. At least have some ideas about if you're a large facility and how to um, get to these items quicker.”
Personal protective equipment	• Using gloves safely • Disposal process of personal protective equipment	• “Like, um, how long it should be used or like when should you change your gloves. We've been, I've had clients ask me that and we change our clients relatively often, but, um, someone just washing their gloves and reusing, for me, that's like I don't think so, but I don't, that may be something to include.”
**Suggestions for future guidance**		
**Theme**	**Examples**	**Quotes**
Communication Material Formats	Format suggestions: • Poster • Flyer • Video • Email blasts • Physical copies • One-pager summarizing guidance	• “It's just not a crisis, or even if they're in a crisis, there's some people that just aren't going to read, you know, materials. But if you put it in the form of video and then we facilitate through the video, it kind of makes it easier. I mean, that, that'd be my suggestion.”
Low literacy	Developing materials with low literacy in mind • Use methods like adding pictures in future guidance to convey message for people with different reading levels	• “… [the guidance should be] at a medium range of- of comprehension” • “You know, we try to make it, you know, put more pictures in there, you know, of how to do things so that it catches everyone. It, it's for everyone at every, you know, level of, uh, intellect, level of understanding.”
Communication Materials Dissemination Partners	Disseminate to existing partnerships and directly to the facility including: • Leaders of facilities • Local health department • Organizations already working with PEH • Hotels and motels PEH may access • Mercy Care • City of Atlanta	• “I think every, every group that has an interaction with individuals in a community. I mean, you go from faith based, uh, groups, because, you know, faith-based group, they don't get this type of information, and in schools. Uh, anyone that's volunteering in a volunteer capacity, that's working with, in any area where they, where they come into contact with a lot of people, but specifically when they're working in environments where individuals are homeless or individuals are marginalized in the community or, um, you know, working in areas of severe poverty.” • “Um, so some things would be helpful is connecting where people already have those connections. So like, the city of Atlanta, their continuum of care partners of home with [name] and [name], there's a phone call every week.”
Trainings	• Web-based training on information presented in the guidance • Q&A sessions with experts to ask for clarification	• “I guess if we had a canned, uh, or like a canned training program that, you know, had a video or something with it that came from let's say the Department of Health or whoever that talked to us and give us a tool to train individuals, a standardized kind of training protocols. Especially in a video kind of, um, a media that we could, you know, sit guys in front of and show 'em, "Hey, these are the recommendations on how to clean, and this is actually how the job should get done."

Since clients were often involved in cleaning and have various levels of reading proficiency, respondents suggested that guidance include pictures. Respondents described a preference for having a web-based training and tools to train staff and clients such as Q&A sessions with experts to ask for clarifications. Respondents suggested that future guidance be communicated through partnerships and systems already in place, such as with shelter leaders, local health departments, organizations that work with PEH (e.g., faith-based organizations), hotels and motels that PEH may use, and Federally Qualified Health Centers.

## Discussion

This project examined cleaning and hand hygiene practices and barriers among staff in homeless shelters and preferred communication strategies. Overall results revealed that shelters engaged in frequent hand hygiene and cleaning, with a specific focus on frequently touched surfaces due to the COVID-19 pandemic. Staff reported insufficient staffing capacity as a barrier to engaging in cleaning and disinfection practices, reinforcing a key challenge reported by other shelters serving PEH prior to and during the pandemic [14, 15]. To maintain cleaning practices in shelters, there was an increased reliance on persons who were not employed in roles that included cleaning duties; in addition to non-maintenance staff and volunteers, clients played a key role in cleaning and disinfection activities. However, frequent client turnover and individual barriers along with access to cleaning supplies presented a challenge to ensuring cleaning was done correctly. Insufficient or ineffective cleaning practices due to these barriers resulted in added burden to staff.

To address staffing challenges, frequent client turnover, and individual barriers, staff identified a need for a range of health education modalities such as trainings, health communication materials, and detailed guidance. High staff turnover in homeless shelters as a reported barrier to adequate training is consistent with a previous study [14]. In another study, researchers found that 57% of homeless shelter staff reported cleaning was part of their regular duties, however of these staff, 43% reported not receiving training on cleaning surfaces for SARS-CoV-2 [11]. Although this project found that most staff reported receiving training, staff turnover was a key barrier to the capacity to train new staff. Additionally, staff were unable to easily use the same training materials they received to train their clients due to formats not being suitable for client training, materials only being offered in English, and not in an accessible literacy level. Shelters need detailed, tailored training materials that can easily be used to train new staff and clients in order to increase knowledge and adherence to recommended guidance.

Respondents also revealed that shelters needed cleaning and disinfection guidance with more specific details such as when and how to clean specific items. Standard guidance from public health partners on hand hygiene and cleaning and disinfection that provides more specificity on supplies, cleaning techniques and best practices, similar to guidance developed specifically for early childhood care and education centers, would be helpful for shelters serving PEH [16]. During outbreaks where specific cleaning activities are needed, guidance could be adapted. Future guidance may also consider how to address potential shortages of cleaning supplies and staff. Since clients play an important role in cleaning and disinfection practices in shelters, guidance should be easily understandable for both staff and clients.

Materials such as trainings or guidance should be developed using best practices for health communication, such as tailoring materials to specific groups served by different shelter types, ensuring materials are linguistically and culturally appropriate, and developing materials that accommodate low literacy levels. During H1N1, it was found that access to many sources of information could be confusing for people in general [17]. However, a best practice from H1N1 response efforts was communication from public health officials to service providers, and availability of information in formats that accommodated low literacy levels, and this should be a standard public health communication practice going forward [17, 18]. When developing materials, it is important that information is presented in an easily digestible manner and can easily be related to clients. For example, respondents suggested the use of visual educational tools such as short, applied videos to help overcome literacy and language barriers; these types of materials could be used routinely to train new clients. In a literature review of the use of visual aids in health education materials, researchers found that people with low literacy had statistically significant improvements in health literacy outcomes when visual aids were developed; the most effective types were pictograms and videos [19]. Using these best communication practices, hand hygiene should continue to be promoted to ensure new clients/staff know the key times to clean hands and the best way to do so. Finally, as some PEH may have physical or mental health problems that would make engaging in cleaning activities difficult, materials can include strategies for how to adapt cleaning practices and protocols to accommodate disabilities or other limitations.

Shelters in this project were able to scale up their hand hygiene and cleaning practices during the COVID-19 pandemic by increasing cleaning frequency and focusing efforts on frequently touched surfaces. Although baseline practices are needed to support general infectious disease prevention efforts, shelters should be prepared to scale up efforts and have sufficient resources to respond to future pandemics or outbreaks. For example, since this project’s completion, the American Rescue Plan Act provided $80,000,000 to health departments to support COVID-19 testing and prevention in homeless service sites, encampments, and other congregate living facilities [20, 21]. Recipients could use the money to support activities such as addressing shelter staff shortages and buying additional supplies [20, 21]. In a study examining the impacts of COVID-19 on PEH and homeless service providers, researchers found that homeless service providers experienced financial challenges such as unexpected expenses related to purchasing PPE and supplies for staff and clients and had to rely on local, non-federal sources of funding at the time of the study [15]. Federal support to help with fiscal and human resources can help to address barriers, develop baseline practices, and plans for scale up. Findings from this project demonstrated the importance of public health and academic partners collaborating with shelter staff to develop educational tools to engage in recommended cleaning and hand hygiene practices.

This assessment has at least two limitations. First, this qualitative assessment was conducted in Atlanta, Georgia, and the perspectives and experiences of the respondents may not be generalizable to other geographic areas. Second, this assessment relied on self-report and did not include an in-person environmental assessment or observational component. As a result, assessment findings could be impacted by response bias.

## Conclusion

This project highlighted that adequate staffing, supplies, and tailored, accessible training materials and guidance are needed to implement cleaning, disinfection, and hygiene interventions; these are critical for preventing and controlling infectious diseases in shelters. Adoption of these strategies at baseline would enhance routine infectious disease prevention, as well as improve public health emergency preparedness for infectious disease outbreaks or pandemics.

## Data Availability

The data that support the findings of this study are available from the authors upon reasonable request (email Candace Rutt at AWR8@CDC.GOV).
